# Supplementary material for: Genetic associations of protein-coding variants in venous thromboembolism
Source: Nat Commun. 2024 Apr 1;15:2819. doi: 10.1038/s41467-024-47178-8 (PMC10984941; doi:10.1038/s41467-024-47178-8)
Supplement: Supplementary file 1 — Supplementary Information [file 41467_2024_47178_MOESM1_ESM.pdf]

Supplementary material content for

Genetic associations of protein-coding variants in venous thromboembolism

Supplementary Note 1 Detailed information on whole-exome sequencing in UKB 2
Supplementary Note 2 Relationship inference and kinship matrix 2
Supplementary Note 3 PRS derivation 2
Figure S1. Manhattan plots and QQ plots for exome-wide gene-level associations of rare coding variants under 12 models 3
Figure S2. Forest plot of sex-stratified sensitivity analysis for gene-level collapsing analysis 5
Figure S3. Manhattan plots for gene-level associations of rare coding variants in female and male 5
Figure S4. Forest plot of ancestry-specific and cross-ancestry meta-analysis sensitivity analysis for gene-level collapsing analysis 6
Figure S5. Effect size (odds ratio) and P-value comparison of significant gene-level associations 6
Figure S6. Leave-one-variant-out (LOVO) analysis for rare variant associations. 7
Figure S7. Manhattan plots and QQ plots for single variant associations of common coding variants in female and male 8
Figure S8. Forest plot of sex-stratified sensitivity analysis for single variant analysis 9
Figure S9. Manhattan plots and QQ plots of ancestry-specific and cross-ancestry meta-analysis sensitivity analysis for single variant associations 10
Figure S10. Forest plot of ancestry-specific and cross-ancestry meta-analysis sensitivity analysis for single variant analysis 11
Figure S11. Effect size (odds ratio) and P-value comparison of significant single variant associations 12
Figure S12. Manhattan plot and QQ plot for single variant analysis of common variants (including both coding and noncoding) 12
Figure S13. Associations between VTE risk genes (or lead SNPs) and 35 selected blood traits 13

Supplementary Note 1 Detailed information on whole-exome sequencing in UKB

Exome sequencing data for 454,988 UKB participants were generated at the Regeneron Genetics Center[1]. Briefly, exomes were captured using the IDT xGen Exome Research Panel v1.0, with the initial 50,000 samples processed with IDT 'lot1' and all others with 'lot2'. Illumina NovaSeq6000 platform was used to sequence the samples at Regeneron Pharmaceuticals with dual-indexed 75×75bp paired-end reads using S2 and S4 flow cells for the initial and subsequent samples respectively, and obtained more than 20x coverage over 95% of targeted bases.

Sequencing data in BCL format was converted to FASTQ format using bcl2fastq v2.19.0, and the assignments of paired-end sequence reads to samples were based on 10-base barcodes. Then initial quality control included sex discordance, contamination, unresolved duplicate sequences, and discordance with microarray genotyping data checks were performed by Regeneron. Finally, a total of 454,796 participants in UKB passed these quality control measures.

Supplementary Note 2 Relationship inference and kinship matrix

A subset of high-quality genetic variants was selected (MAF > 0.1%, missingness < 1%, Hardy–Weinberg equilibrium (HWE) $P > 10^{-6}$ and two waves of pruning using --indep-pairwise 200 100 0.1 and --indep-pairwise 200 100 0.05) for the relationship inference. King software was used to calculate pairwise heterozygote concordance rates and the kinship coefficient using high-quality variants. Then we removed related participants using the kinship coefficient threshold at 0.0884 (--kinship function), which denoted the second-degree relatives or closer. For pairs exhibiting a kinship coefficient greater than 0.0884, we first iteratively excluded samples that showed relationships with multiple individuals until no such samples remained. We then randomly removed one of the remaining pairs.

Supplementary Note 3 PRS derivation

Genome-wide genotype data after imputation were available for 488 377 individuals in UKB, with details of DNA acquisition, sample manipulation, and quality control (QC) described in previous literature [2]. In addition to sample QC performed by the UKB team, we further cleaned the genotype data based on QC metrics at the individual sample and SNP levels as in previous study [3].

Summary statistics from the largest existing GWAS meta-analysis without the UKB (from CHB-CVDC/DBDS, Intermountain healthcare, and deCODE) were used as target dataset [4], which contains a total of 57,467 cases and 1,006,954 controls, to create a PRS for VTE. We first derived the weights for each SNP by PRS–CS, which utilizes a Bayesian regression-based algorithm to model linkage disequilibrium from an external reference set and places a continuous shrinkage prior to SNP effect sizes. We used 1000 Genomes European ancestry as LD reference and auto option in PRS–CS. Then the resulting weights were used to calculate the sum of valid per-allele scores using the 'score' function with the sum option in PLINK v.2.00.

Figure S1. Manhattan plots and QQ plots for exome-wide gene-level associations of rare coding variants under 12 models

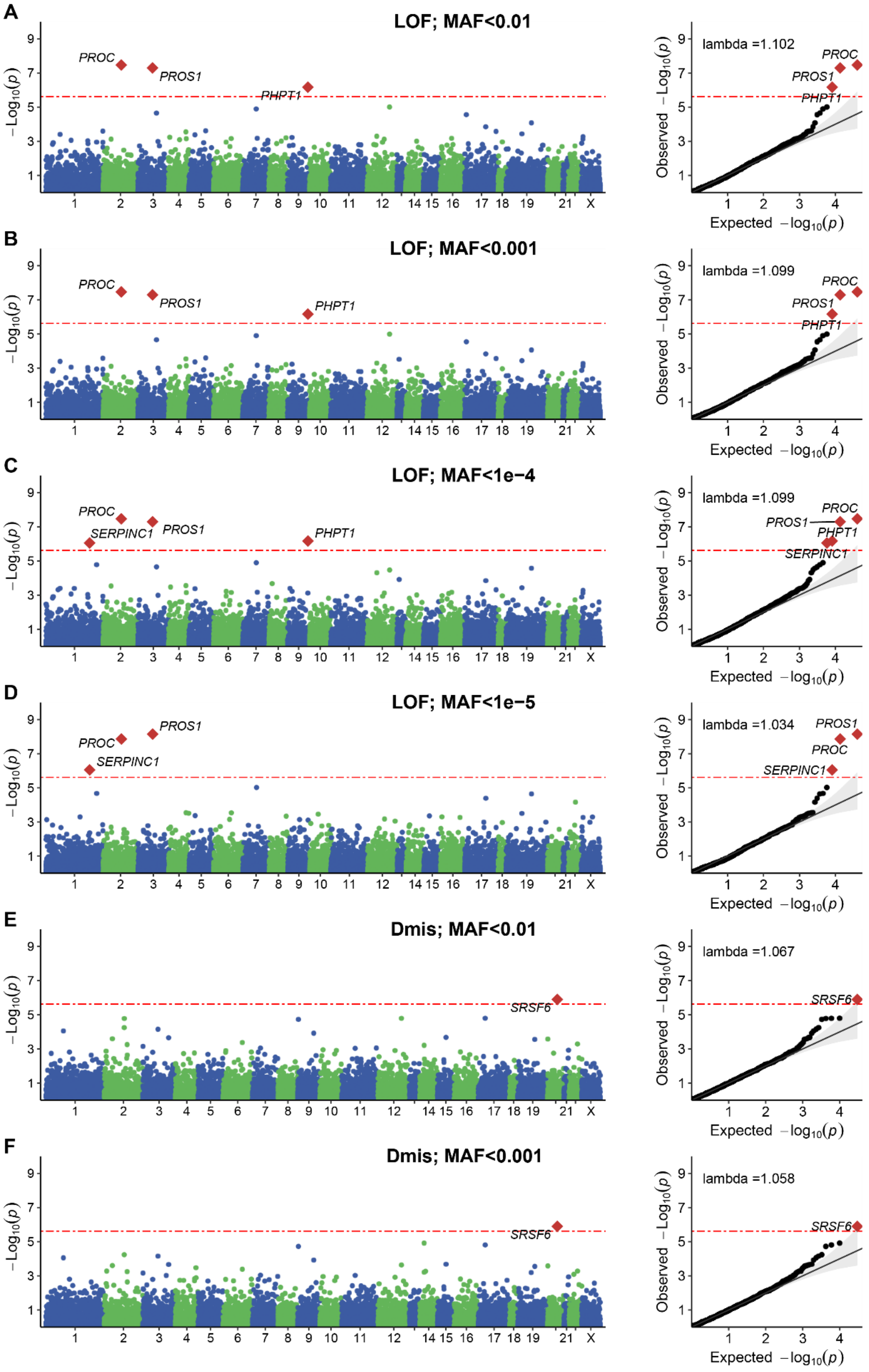

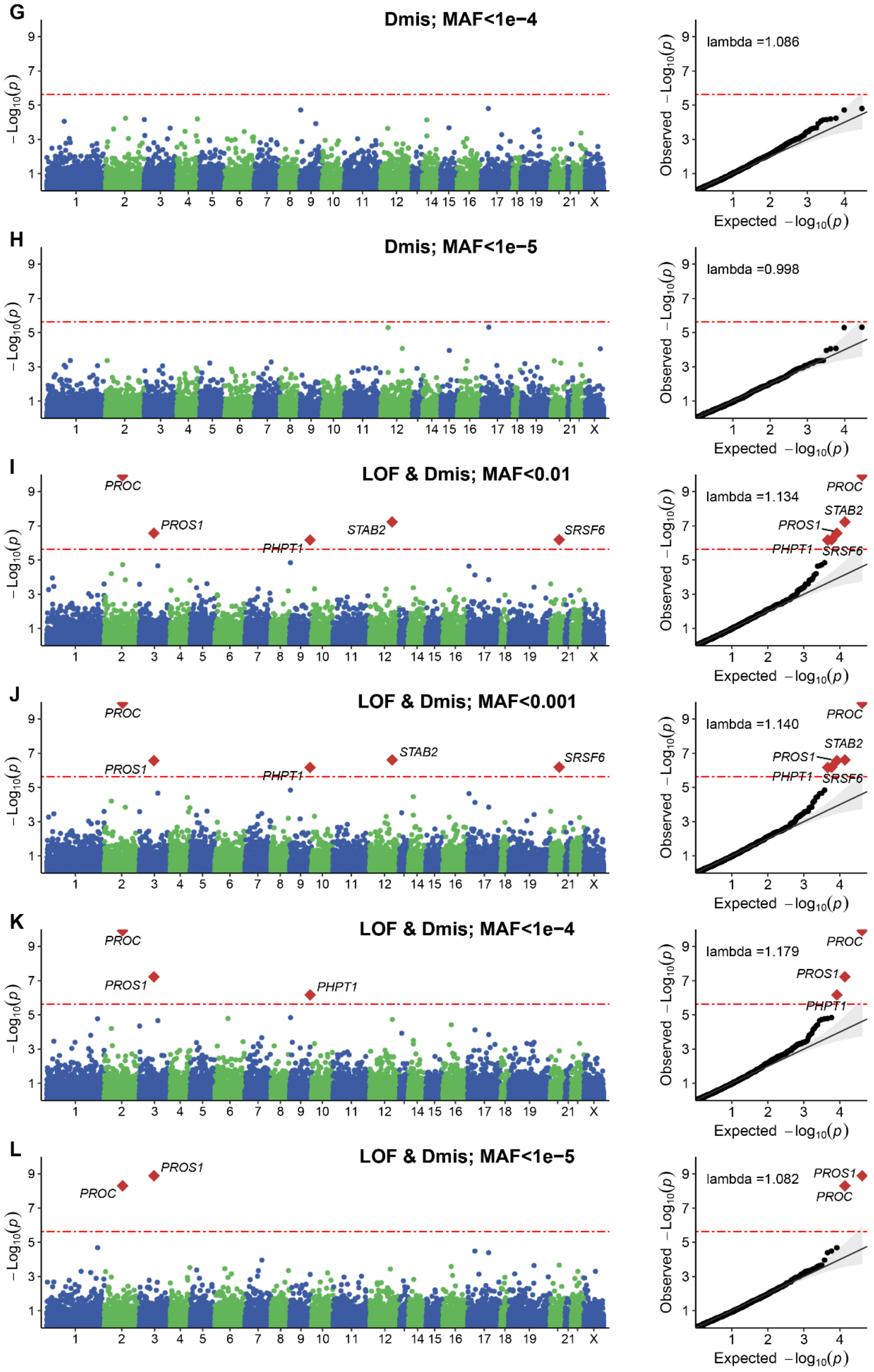

Left panel: Manhattan plots of results from gene-level association analysis in the 14,723 VTE cases and 334,315 control subjects. The colors on the plots show the delimitation of chromosomes.

Right panel: QQ plots of results from gene-level association analysis. The y-axis represents the observed -$\log_{10}$(p-value), while the x-axis represents the expected under the null-hypothesis. Across all 12 models, no systemic inflation is observed.

P values shown are two-sided and Bonferroni correction were used. The red dotted horizontal line represents the Bonferroni significance threshold (P=2.42×10^{-6}).

Figure S2. Forest plot of sex-stratified sensitivity analysis for gene-level collapsing analysis

Gene	Sex	Variant type; Frequency cutoff	OR (95% CI)	P−value
PHPT1	All	LOF; MAF<0.001	1.08 (1.05~1.11)	6.8e−7
	Female	LOF; MAF<0.001	1.10 (1.06~1.14)	3.7e−6
	Male	LOF; MAF<0.001	1.06 (1.00~1.12)	0.023
PROC	All	LOF & Dmis; MAF<0.001	1.07 (1.05~1.10)	1.2e−10
	Female	LOF & Dmis; MAF<0.001	1.08 (1.04~1.12)	3.8e−6
	Male	LOF & Dmis; MAF<1e−5	1.14 (1.07~1.20)	1.1e−5
PROS1	All	LOF & Dmis; MAF<1e−5	1.08 (1.06~1.11)	1.3e−9
	Female	LOF & Dmis; MAF<1e−5	1.10 (1.06~1.14)	2.6e−6
	Male	LOF & Dmis; MAF<0.001	1.03 (1.02~1.04)	4.8e−6
SERPINC1	All	LOF; MAF<1e−4	1.19 (1.11~1.28)	8.9e−7
	Female	LOF; MAF<1e−4	1.22 (1.10~1.35)	0.00019
	Male	LOF; MAF<1e−4	1.18 (1.07~1.30)	0.00095
SRSF6	All	LOF & Dmis; MAF<0.001	1.04 (1.02~1.06)	6.6e−7
	Female	Dmis; MAF<0.001	1.04 (1.01~1.06)	0.0024
	Male	LOF & Dmis; MAF<0.001	1.05 (1.03~1.07)	3.8e−5
STAB2	All	LOF & Dmis; MAF<0.01	1.01 (1.01~1.02)	6e−8
	Female	LOF & Dmis; MAF<0.001	1.01 (1.00~1.02)	0.00036
	Male	LOF & Dmis; MAF<0.01	1.02 (1.01~1.02)	3.7e−6

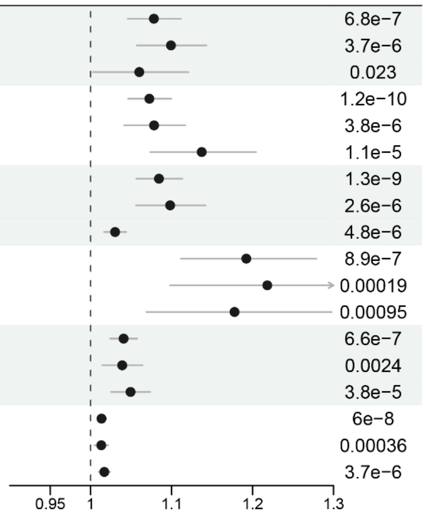

The forest plot shows the results of sex-stratified sensitivity analysis for significant genes identified in the gene-level collapsing test in all participants (14,723 VTE cases and 334,315 controls), female (7,629 VTE cases and 180,090 controls) and male (7,094 VTE cases and 154,225 controls). All models were adjusted for age, sex (except for sex-stratified analysis), and top ten ancestral principal components (PCs). *P* values shown are two-sided and no adjustments were made for multiple comparisons.

Figure S3. Manhattan plots for gene-level associations of rare coding variants in female and male

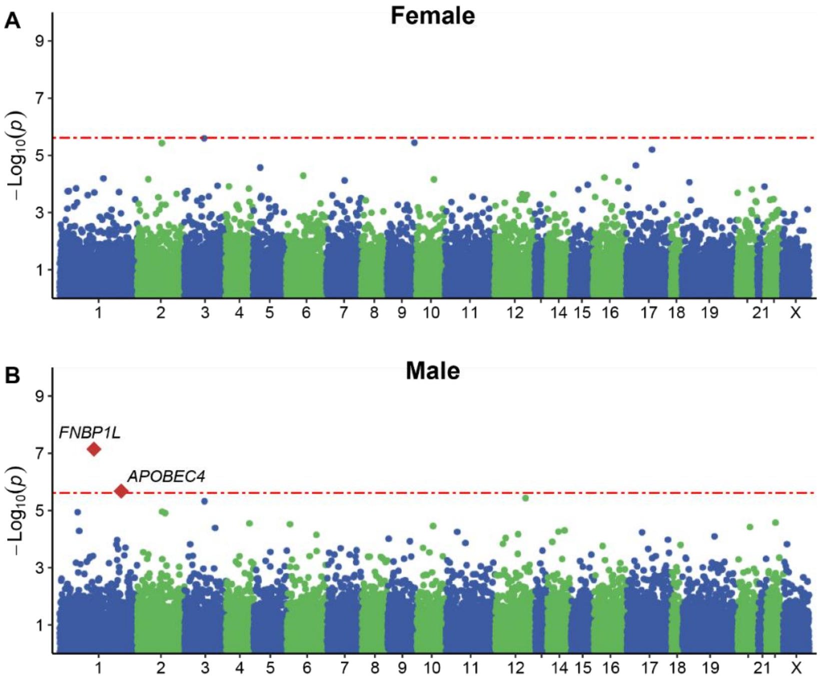

Manhattan plot of results from gene-level association analysis in female (A, 7,629 VTE cases and 180,090 controls); and male (B, 7,094 VTE cases and 154,225 controls). The colors on the plots show the delimitation of chromosomes. *P* values shown are two-sided and Bonferroni correction were used. The red dotted horizontal line represents the Bonferroni significance threshold (P=2.42×10^{-6}).

Figure S4. Forest plot of ancestry-specific and cross-ancestry meta-analysis sensitivity analysis for gene-level collapsing analysis

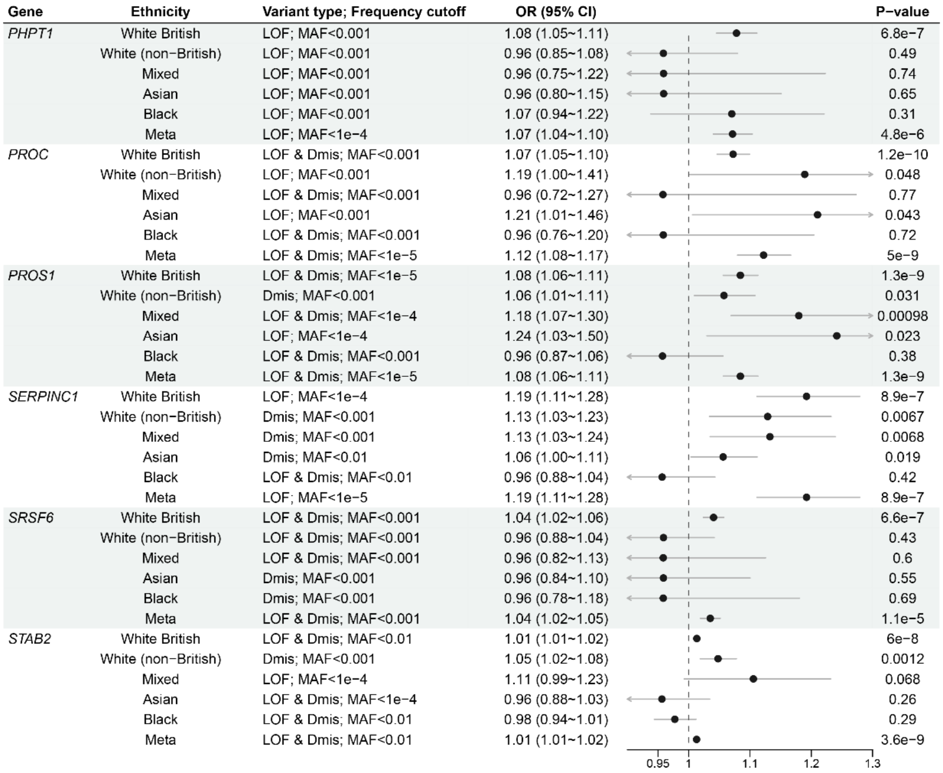

The forest plot shows the results of ancestry-specific and cross-ancestry meta-analysis sensitivity for significant genes identified in the gene-level collapsing test. All models were adjusted for age, sex, and top ten ancestral principal components (PCs). *P* values shown are two-sided and no adjustments were made for multiple comparisons.

Figure S5. Effect size (odds ratio) and P-value comparison of significant gene-level associations

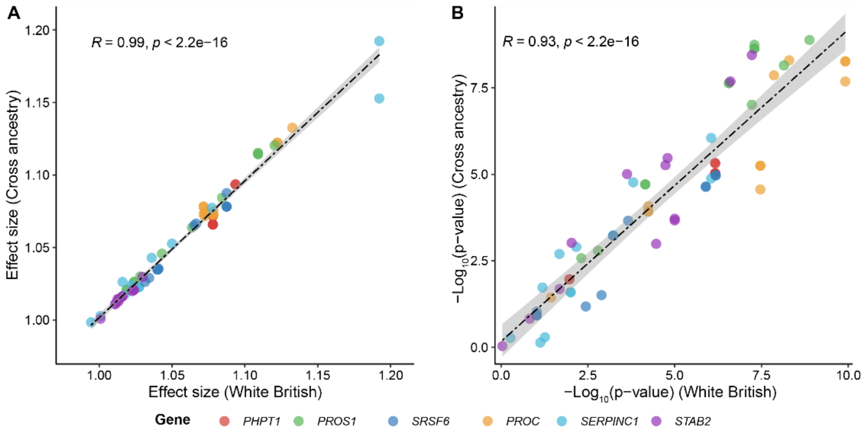

(A) Effect size (odds ratio) comparison between cross-ancestry meta-analysis (ancestry specific analysis followed by meta-analysis) and White British only. **(B)** P-value comparison between cross-ancestry meta-analysis (ancestry specific analysis followed by meta-analysis) and White British only. Pearson correlation (R) estimates and two-sided *P* values are shown.

Figure S6. Leave-one-variant-out (LOVO) analysis for rare variant associations.

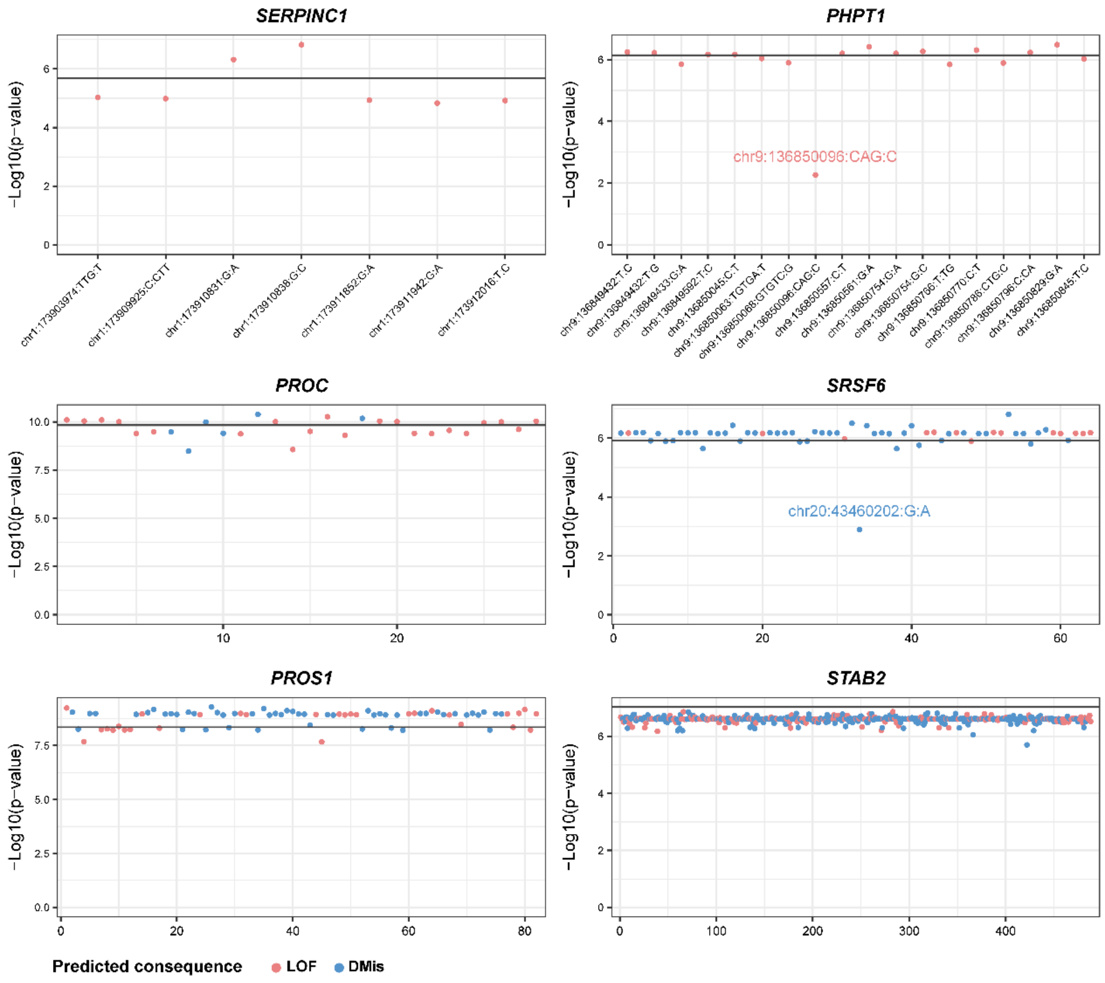

The x-axis represents the single variant removed from the gene-level collapsing analysis, while the y-axis shows the -$\log_{10}$(p-value) of the association without that given variant. P-values were obtained from SKAT-O tests using logistic mixed effects models implemented in SAGE-GENE+, adjusting for sex, age, and 10 top principal components (PCs). *P* values shown are two-sided and no adjustments were made for multiple comparisons. The black horizontal line shows the original result without any variant removed. Variants are annotated with the variant name in format.

Figure S7. Manhattan plots and QQ plots for single variant associations of common coding variants in female and male

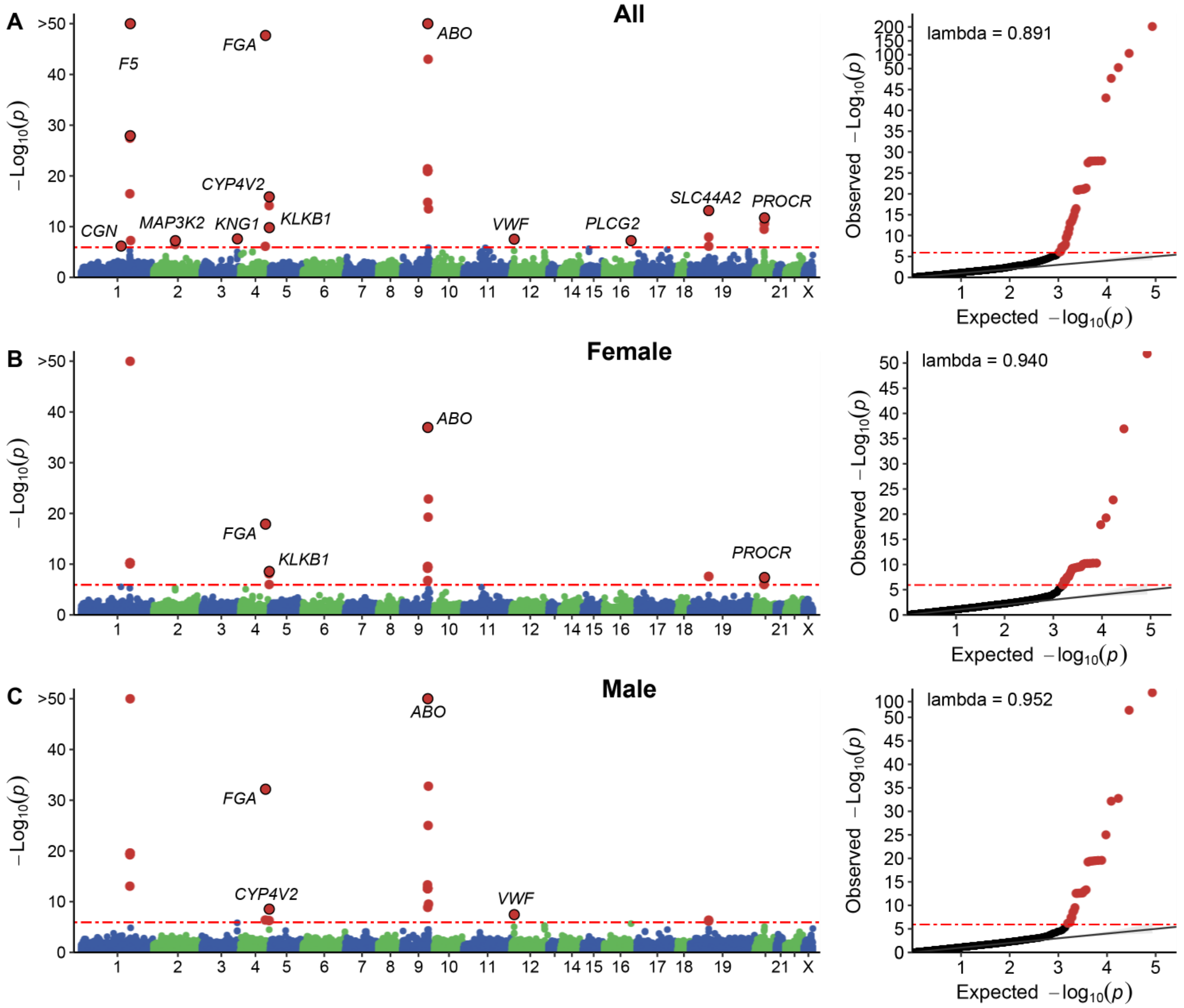

Left panel: Manhattan plots of results from single variant analysis for common coding variants in all participants (**A**, 14,723 VTE cases and 334,315 controls), female (**B**, 7,629 VTE cases and 180,090 controls) and male (**C**, 7,094 VTE cases and 154,225 controls). The colors on the plots show the delimitation of chromosomes. Each lead SNP (red dots with black border) was annotated with its corresponding gene name. The y-axis is capped at 50.

Right panel: QQ plots of results from single variant analysis for common coding variants. The y-axis represents the observed -$\log_{10}$(p-value), while the x-axis represents the expected under the null-hypothesis. No systemic inflation is observed.

P values shown are two-sided and Bonferroni correction were used. The red dotted horizontal line represents the significance threshold ($P=1.17\times10^{-6}$, Bonferroni correction for 42,911 common coding variants).

Figure S8. Forest plot of sex-stratified sensitivity analysis for single variant analysis

Gene	ID	Ethnicity	OR (95% CI)	P−value
ABO	chr9:133257521:T:TC	All	1.31 (1.28~1.34)	6.9e−106
		Female	1.25 (1.20~1.29)	1.2e−37
		Male	1.38 (1.33~1.42)	1.5e−73
CGN	chr1:151525667:G:A	All	0.76 (0.68~0.84)	6.8e−7
		Female	0.68 (0.58~0.80)	3.1e−6
		Male	0.84 (0.72~0.97)	0.022
CYP4V2	chr4:186201165:T:G	All	0.90 (0.88~0.92)	1.4e−16
		Female	0.90 (0.87~0.93)	8.6e−9
		Male	0.90 (0.86~0.93)	3e−9
F5	chr1:169542882:G:A	All	0.86 (0.83~0.88)	1.2e−28
		Female	0.88 (0.85~0.92)	6.2e−11
		Male	0.83 (0.80~0.86)	2.8e−20
	chr1:169549811:C:T	All	2.38 (2.25~2.52)	2e−202
		Female	2.16 (2.00~2.35)	1.2e−78
		Male	2.63 (2.43~2.85)	1.2e−129
FGA	chr4:154586438:T:C	All	1.21 (1.18~1.24)	2.1e−48
		Female	1.18 (1.13~1.22)	1.3e−18
		Male	1.25 (1.21~1.30)	6.9e−33
KLKB1	chr4:186258056:T:C	All	0.92 (0.90~0.94)	1.6e−10
		Female	0.92 (0.88~0.95)	1.1e−6
		Male	0.92 (0.89~0.96)	3.5e−5
KNG1	chr3:186725229:T:C	All	0.94 (0.91~0.96)	2.6e−8
		Female	0.95 (0.92~0.98)	0.002
		Male	0.92 (0.89~0.95)	1.5e−6
MAP3K2	chr2:127322230:A:G	All	1.07 (1.05~1.10)	5.9e−8
		Female	1.08 (1.05~1.12)	5.2e−6
		Male	1.06 (1.02~1.10)	0.0019
PLCG2	chr16:81854547:A:G	All	1.07 (1.04~1.10)	5.9e−8
		Female	1.05 (1.02~1.09)	0.003
		Male	1.09 (1.05~1.13)	2.2e−6
PROCR	chr20:35176751:A:G	All	1.15 (1.11~1.20)	1.9e−12
		Female	1.16 (1.10~1.23)	4.3e−8
		Male	1.14 (1.08~1.20)	8.7e−6
SLC44A2	chr19:10631494:A:G	All	0.90 (0.87~0.92)	6.9e−14
		Female	0.89 (0.86~0.93)	2.7e−8
		Male	0.90 (0.86~0.94)	4.5e−7
VWF	chr12:6044368:T:C	All	1.07 (1.04~1.10)	3e−8
		Female	1.04 (1.01~1.08)	0.017
		Male	1.10 (1.06~1.14)	3.6e−8

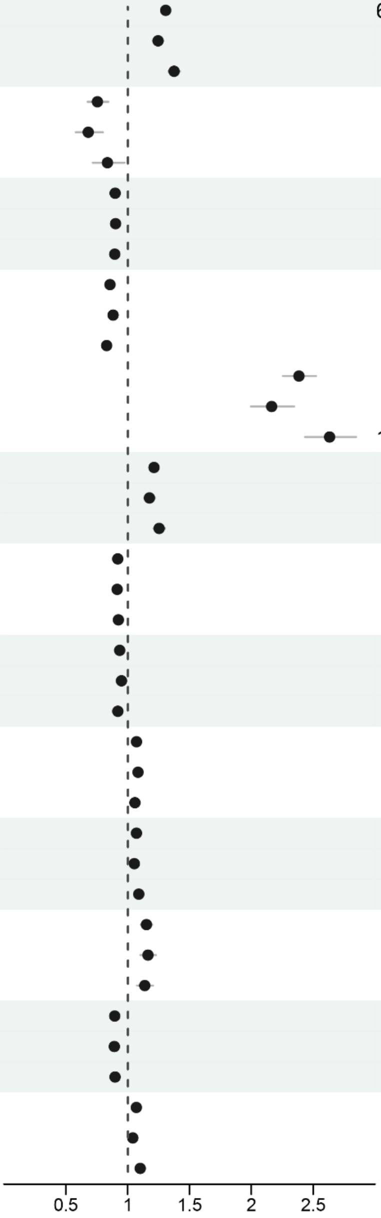

The forest plot shows the results of sex-stratified sensitivity analysis for significant lead SNPs identified from the single variant test in all participants (14,723 VTE cases and 334,315 controls), female (7,629 VTE cases and 180,090 controls) and male (7,094 VTE cases and 154,225 controls). All models were adjusted for age, sex (except for sex-stratified analysis), and top ten ancestral principal components (PCs). *P* values shown are two-sided and no adjustments were made for multiple comparisons.

Figure S9. Manhattan plots and QQ plots of ancestry-specific and cross-ancestry meta-analysis sensitivity analysis for single variant associations

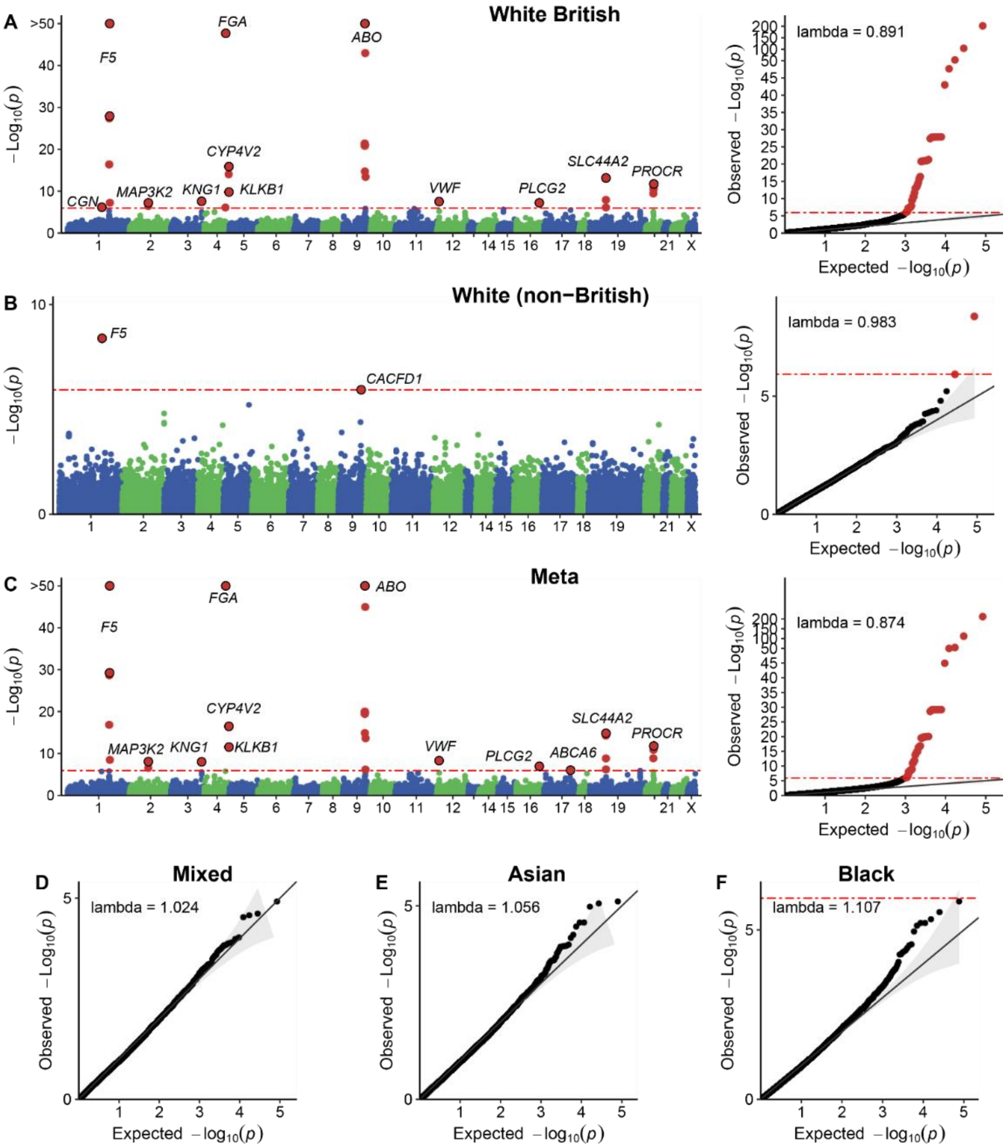

(A, B, C) Left panel: Manhattan plots of results from single variant analysis for common coding variants in White British (**A**, 14,723 VTE cases and 334,315 controls), non-British White population (**B**, 936 VTE cases and 24,735 controls) and cross-ancestry meta-analysis (**C**). The colors on the plots show the delimitation of chromosomes. Each lead SNP (red dots with black border) was annotated with its corresponding gene name. There were no significant results in other ethnicity (Manhattan plots not shown).

(A, B, C) Right panel, D, E, F : QQ plots of results from single variant analysis for common coding variants. The y-axis represents the observed -$\log_{10}$(p-value), while the x-axis represents the expected under the null-hypothesis. No systemic inflation is observed.

P values shown are two-sided and Bonferroni correction were used. The red dotted horizontal line represents the significance threshold ($P=1.17\times10^{-6}$, Bonferroni correction for 42,911 common coding variants).

Figure S10. Forest plot of ancestry-specific and cross-ancestry meta-analysis sensitivity analysis for single variant analysis

The forest plot shows the results of ancestry-specific and cross-ancestry meta-analysis sensitivity analysis for significant lead SNPs identified in the single variant test. All models were adjusted for age, sex (except for sex-stratified analysis), and top ten ancestral principal components (PCs). *P* values shown are two-sided and no adjustments were made for multiple comparisons.

Gene	ID	Ethnicity	OR (95% CI)	P−value
ABO	chr9:133257521:T:TC	White British	1.31 (1.28~1.34)	6.9e−106
		White (non−British)	1.23 (1.11~1.35)	4e−5
		Mixed	1.37 (1.10~1.69)	0.0043
		Asian	1.30 (1.06~1.59)	0.011
		Black	1.26 (1.04~1.52)	0.016
		Meta	1.30 (1.27~1.33)	1.4e−113
CGN	chr1:151525667:G:A	White British	0.76 (0.68~0.84)	6.8e−7
		White (non−British)	1.03 (0.68~1.56)	0.88
		Mixed	1.12 (0.28~4.56)	0.87
		Asian		
		Black	0.74 (0.09~5.87)	0.77
		Meta	0.77 (0.69~0.86)	2.1e−6
CYP4V2	chr4:186201165:T:G	White British	0.90 (0.88~0.92)	1.4e−16
		White (non−British)	0.87 (0.78~0.96)	0.0053
		Mixed	0.93 (0.75~1.15)	0.49
		Asian	1.08 (0.88~1.32)	0.48
		Black	1.05 (0.87~1.25)	0.63
		Meta	0.90 (0.88~0.92)	3.2e−17
F5	chr1:169542882:G:A	White British	0.86 (0.83~0.88)	1.2e−28
		White (non−British)	0.86 (0.77~0.96)	0.0064
		Mixed	0.89 (0.69~1.14)	0.36
		Asian	0.97 (0.78~1.21)	0.79
		Black	0.98 (0.77~1.23)	0.83
		Meta	0.86 (0.84~0.88)	6e−30
	chr1:169549811:C:T	White British	2.38 (2.25~2.52)	2e−202
		White (non−British)	2.04 (1.61~2.59)	4.1e−9
		Mixed	1.59 (0.84~3.00)	0.16
		Asian	2.67 (1.58~4.53)	0.00027
		Black	0.77 (0.10~5.85)	0.8
		Meta	2.36 (2.24~2.49)	2.6e−212
FGA	chr4:154586438:T:C	White British	1.21 (1.18~1.24)	2.1e−48
		White (non−British)	1.21 (1.09~1.34)	0.00045
		Mixed	1.07 (0.86~1.35)	0.54
		Asian	1.11 (0.90~1.38)	0.32
		Black	1.18 (0.98~1.41)	0.082
		Meta	1.21 (1.18~1.24)	1.2e−51
KLKB1	chr4:186258056:T:C	White British	0.92 (0.90~0.94)	1.6e−10
		White (non−British)	0.85 (0.77~0.94)	0.0023
		Mixed	0.82 (0.66~1.03)	0.092
		Asian	0.98 (0.80~1.19)	0.81
		Black	1.01 (0.84~1.21)	0.92
		Meta	0.92 (0.89~0.94)	2.9e−12
KNG1	chr3:186725229:T:C	White British	0.94 (0.91~0.96)	2.6e−8
		White (non−British)	0.98 (0.89~1.08)	0.66
		Mixed	0.82 (0.66~1.01)	0.067
		Asian	1.02 (0.83~1.24)	0.86
		Black	0.88 (0.73~1.06)	0.17
		Meta	0.94 (0.92~0.96)	9.5e−9
MAP3K2	chr2:127322230:A:G	White British	1.07 (1.05~1.10)	5.9e−8
		White (non−British)	1.07 (0.97~1.18)	0.19
		Mixed	0.97 (0.77~1.22)	0.77
		Asian	1.05 (0.85~1.31)	0.65
		Black	1.24 (1.02~1.51)	0.029
		Meta	1.07 (1.05~1.10)	8.5e−9
PLCG2	chr16:81854547:A:G	White British	1.07 (1.04~1.10)	5.9e−8
		White (non−British)	1.01 (0.91~1.12)	0.87
		Mixed	1.08 (0.85~1.37)	0.54
		Asian	1.16 (0.93~1.45)	0.18
		Black	0.80 (0.60~1.06)	0.12
		Meta	1.07 (1.04~1.09)	1.1e−7
PROCR	chr20:35176751:A:G	White British	1.15 (1.11~1.20)	1.9e−12
		White (non−British)	1.15 (0.98~1.35)	0.08
		Mixed	0.75 (0.51~1.10)	0.14
		Asian	1.14 (0.88~1.46)	0.32
		Black	0.95 (0.68~1.32)	0.77
		Meta	1.14 (1.10~1.19)	1.6e−12
SLC44A2	chr19:10631494:A:G	White British	0.90 (0.87~0.92)	6.9e−14
		White (non−British)	0.83 (0.74~0.93)	0.0013
		Mixed	1.04 (0.79~1.36)	0.8
		Asian	0.95 (0.75~1.21)	0.68
		Black	0.94 (0.62~1.43)	0.76
		Meta	0.89 (0.87~0.92)	1.5e−15
VWF	chr12:6044368:T:C	White British	1.07 (1.04~1.10)	3e−8
		White (non−British)	1.09 (0.99~1.19)	0.091
		Mixed	1.08 (0.87~1.35)	0.46
		Asian	1.04 (0.82~1.31)	0.74
		Black	1.04 (0.87~1.25)	0.65
		Meta	1.07 (1.05~1.10)	5.1e−9

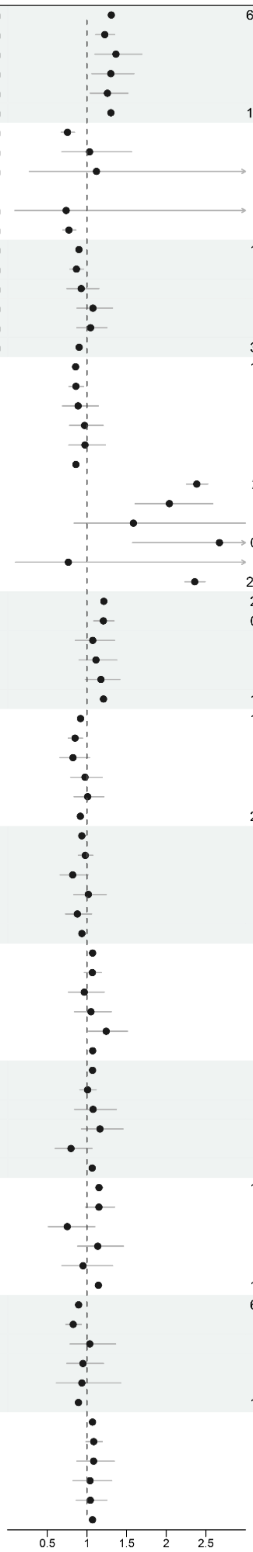

Figure S11. Effect size (odds ratio) and P-value comparison of significant single variant associations

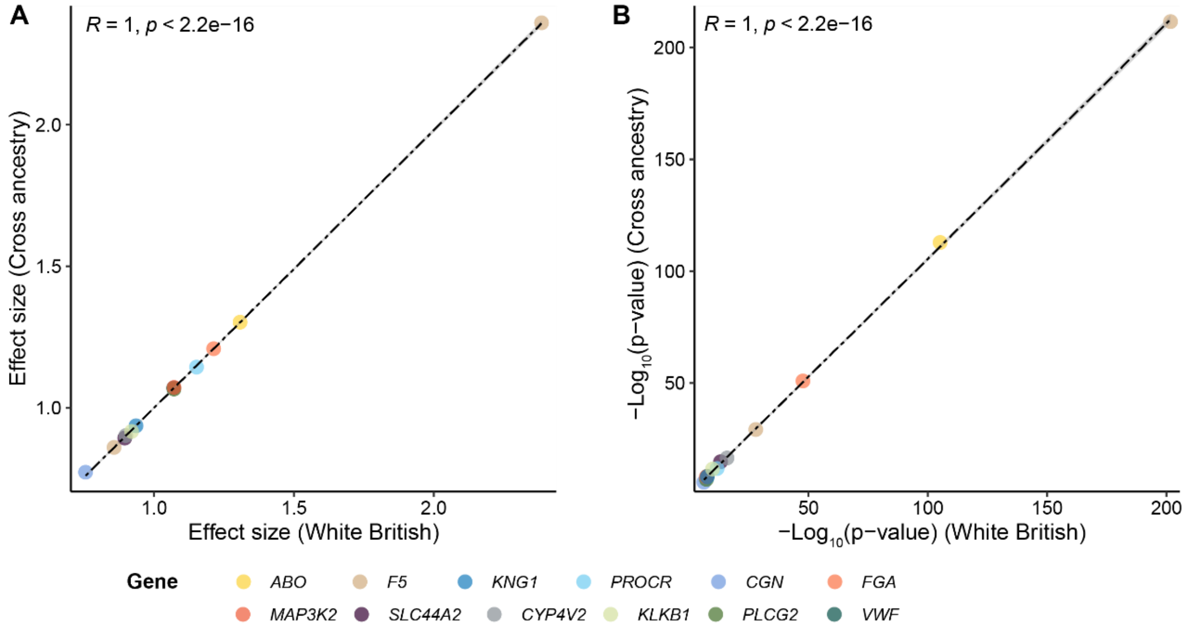

(A) Effect size (odds ratio) comparison between cross-ancestry meta-analysis (ancestry specific analysis followed by meta-analysis) and White British only.

(B) P-value comparison between cross-ancestry meta-analysis (ancestry specific analysis followed by meta-analysis) and White British only.

Pearson correlation (R) estimates and two-sided *P* values are shown. Each lead SNP was annotated with its corresponding gene name.

Figure S12. Manhattan plot and QQ plot for single variant analysis of common variants (including both coding and noncoding)

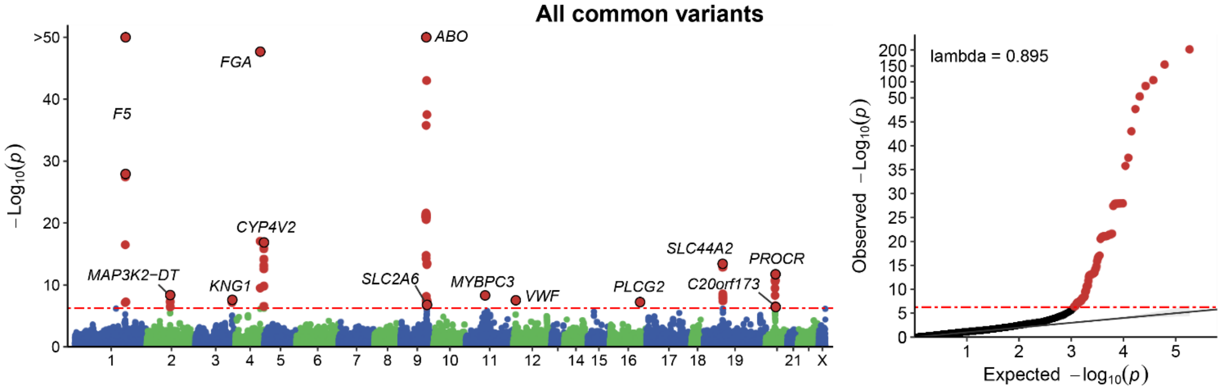

Manhattan plot and QQ plot of results from single variant analysis for common variants (including both coding and noncoding) in the 14,723 VTE cases and 334,315 control subjects. *P* values shown are two-sided and Bonferroni correction were used. The red dotted horizontal line represents the significance threshold ($P=5.38\times10^{-7}$, Bonferroni correction for 92,895 common variants). The colors on the plots show the delimitation of chromosomes. Each lead SNP (red dots with black border) was annotated with its corresponding gene name. The y-axis is capped at 50.

Figure S13. Associations between VTE risk genes (or lead SNPs) and 35 selected blood traits

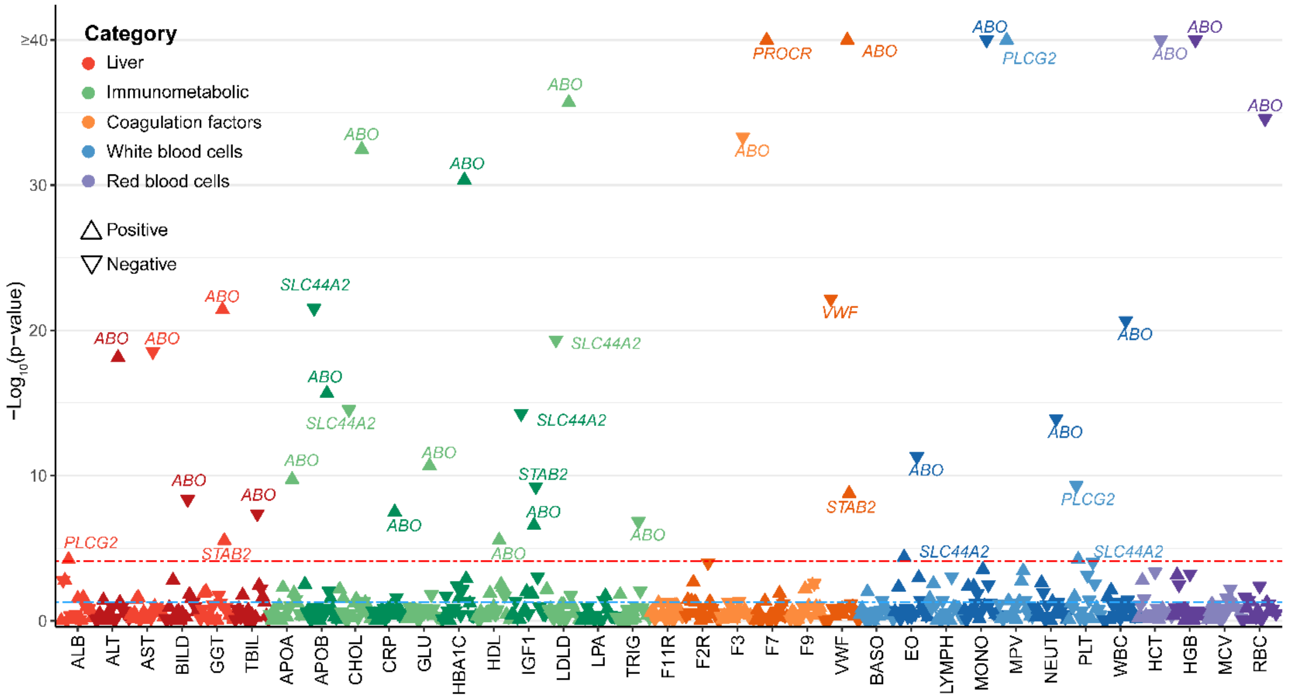

Multiple-trait Manhattan plot representing the association results between VTE risk genes (or lead SNPs) and 35 selected blood traits. Phenotypes are labeled on the x-axis. *P* values shown are two-sided and Bonferroni correction were used. The dashed line represents Bonferroni-corrected significance for 6 risk genes, 13 associated lead SNPs and 35 traits ($P<7.51\times10^{-5}$). Arrows pointing upwards and downwards indicate an increase or decrease in a trait, respectively. Each lead SNP (red dots with black border)was annotated with its corresponding gene name. The y-axis is capped at 40.

Abbreviations: RBC, Red blood cell count; HGB, hemoglobin concentration; HCT, Hematocrit percentage; MCV, Mean corpuscular volume; WBC, White blood cell count; BASO, Basophil count; EO, Eosinophil count; MONO, Monocyte count; NEUT, Neutrophil count; LYMPH, Lymphocyte count; MPV, Mean platelet volume; PLT, Platelet count; APOA, Apolipoprotein A; APOB, Apolipoprotein B; CHOL, Cholesterol; CRP, C-reactive protein; GLU, Glucose; HBA1C, Glycated hemoglobin; LDLD, LDL direct; LPA, Lipoprotein A; TRIG, Triglycerides; IGF1, IGF-1; ALB, Albumin; ALT, Alanine aminotransferase; AST, Aspartate aminotransferase; BILD, Direct bilirubin; GGT, Gamma glutamyltransferase; TBIL, Total bilirubin; F11R, Junctional adhesion molecule A; F2R, Proteinase-activated receptor 1; F3, Tissue factor; F7, Coagulation factor VII; F9, Coagulation factor IX; VWF, von Willebrand factor.

References:

1 Van Hout, C. V. *et al.* Exome sequencing and characterization of 49,960 individuals in the UK Biobank. *Nature* **586**, 749-756, doi:10.1038/s41586-020-2853-0 (2020).

2 Bycroft, C. *et al.* The UK Biobank resource with deep phenotyping and genomic data. *Nature* **562**, 203-209, doi:10.1038/s41586-018-0579-z (2018).

3 He, X. Y. *et al.* Association between polygenic risk for Alzheimer's disease and brain structure in children and adults. *Alzheimers Res Ther* **15**, 109, doi:10.1186/s13195-023-01256-z (2023).

4 Ghouse, J. *et al.* Genome-wide meta-analysis identifies 93 risk loci and enables risk prediction equivalent to monogenic forms of venous thromboembolism. *Nat Genet* **55**, 399-409, doi:10.1038/s41588-022-01286-7 (2023).
