# Supplementary material for: Genetic associations of protein-coding variants in venous thromboembolism
Source: Nat Commun. 2024 Apr 1;15:2819. doi: 10.1038/s41467-024-47178-8 (PMC10984941; doi:10.1038/s41467-024-47178-8)
Supplement: Supplementary file 3 — Description of Additional Supplementary Files [file 41467_2024_47178_MOESM3_ESM.pdf]

Description of Additional Supplementary Files

File Name: Supplementary Data 1
Description: Detailed VTE outcome definition.

File Name: Supplementary Data 2
Description: Baseline characteristics of the study participants by VTE case-control status. Values are median (inter-quartile range) or numbers (percentage). P values are derived using either Student's t-test or Chi-square test. P values shown are two-sided and no adjustments were made for multiple comparisons.
Abbreviations: IQR, inter-quartile range; RBC, Red blood cell (erythrocyte) count; HGB, hemoglobin concentration; HCT, Hematocrit percentage; MCV, Mean corpuscular volume; WBC, White blood cell (erythrocyte) count; BASO, Basophil count; EO, Eosinophil count; MONO, Monocyte count; NEUT, Neutrophil count; LYMPH, Lymphocyte count; MPV, Mean platelet (thrombocyte) volume; PLT, Platelet count; APOA, Apolipoprotein A; APOB, Apolipoprotein B; CHOL, Cholesterol; CRP, C-reactive protein; GLU, Glucose; HBA1C, Glycated hemoglobin (HbA1c); HDL, HDL cholesterol; LDLD, LDL direct; LPA, Lipoprotein A; TRIG, Triglycerides; IGF1, IGF-1; ALB, Albumin; ALT, Alanine aminotransferase; AST, Aspartate aminotransferase; BILD, Direct bilirubin; GGT, Gamma glutamyltransferase; TBIL, Total bilirubin; F11R, Junctional adhesion molecule A; F2R, Proteinase-activated receptor 1; F3, Tissue factor; F7, Coagulation factor VII; F9, Coagulation factor IX; VWF, von Willebrand factor.

File Name: Supplementary Data 3
Description: Details of 12 collapsing models. For rare variants with MAF<0.01, a gene-level collapsing analysis was performed. We included 12 models to qualify variant criteria for 20,629 genes, which vary in terms of MAF ($<10^{-5}$, $<10^{-4}$, $<10^{-3}$, and$<10^{-2}$), and predicted consequence (LOF, Dmis, and LOF-plus-Dmis). rare variants: variants with MAF<0.01 that are not ultra-rare with MAC>10; Ultra-rare variants: variants with MAC≤10.

File Name: Supplementary Data 4
Description: Significant gene associations with VTE in the gene-level collapsing analysis (across all 12 models). P values shown are two-sided and no adjustments were made for multiple comparisons.
Region: gene name; Group: annotation mask; max_MAF: maximum MAF cutoff; Pvalue: p value for SKAT-O test; Pvalue_Burden: p value for BURDEN test; Pvalue_SKAT: p value for SKAT test; BETA_Burden: effect size of gene-level collapsing analysis; SE_Burden: standard error of BETA_Burden; MAC: minor allele count in the set; MAC_case: minor allele count in cases; MAC_control: minor allele count in controls; Number_rare: number of markers that are not ultra-rare with MAC>10; Number_ultra_rare: number of markers that are ultra-rare with MAC ≤10.

File Name: Supplementary Data 5
Description: Top 10 gene associations with VTE in each model. P values shown are two-sided and no adjustments were made for multiple comparisons.
Region: gene name; Group: annotation mask; max_MAF: maximum MAF cutoff; Pvalue: p value for SKAT-O test; Pvalue_Burden: p value for BURDEN test; Pvalue_SKAT: p value for SKAT test; BETA_Burden: effect size of gene-level collapsing analysis; SE_Burden: standard error of BETA_Burden; MAC: minor allele count in the set; MAC_case: minor allele count in cases; MAC_control: minor allele count in controls; Number_rare: number of markers that are not ultra-rare with MAC>10; Number_ultra_rare: number of markers that are ultra-rare with MAC ≤10.

File Name: Supplementary Data 6
Description: Top 3 gene-level associations with VTE in the sensitivity analysis (sex-stratified and ancestry-specific). P values shown are two-sided and no adjustments were made for multiple comparisons. Significant associations were highlighted in red.
Region: gene name; Group: annotation mask; max_MAF: maximum MAF cutoff; Pvalue: p value for SKAT-O test; Pvalue_Burden: p value for BURDEN test; Pvalue_SKAT: p value for SKAT test; BETA_Burden: effect size of gene-level collapsing analysis; SE_Burden: standard error of BETA_Burden; MAC: minor allele count in the set; MAC_case: minor allele count in cases; MAC_control: minor allele count in controls; Number_rare: number of markers that are not ultra-rare with MAC>10; Number_ultra_rare: number of markers that are ultra-rare with MAC ≤10.

File Name: Supplementary Data 7
Description: Carrier frequency of 6 VTE-associated genes and VTE prevalence in QV carriers within each gene.

File Name: Supplementary Data 8
Description: Burden heritability estimates VTE in UK Biobank unrelated caucasian samples.
bhr_h2: estimate of burden heritability; bhr_h2_se: standard error of burden heritability estimate; n_significant_genes: number of exome-wide significant genes for VTE in the frequency-function group based on chi-squared significance threshold; fraction_h2_significant_genes: fraction of burden heritability explained by exome-wide significant genes for VTE in the frequency-function group; fraction_h2_significant_genes_se: standard error of the fraction of burden heritability explained by exome-wide significant genes for VTE in the frequency-function group; intercept: intercept from the BHR model; intercept_se: standard error of the intercept from the BHR model; lambda_gc: the genomic inflation factor, estimated as the median gene burden chi-squared statistic divided by the expected median of the chi-squared distribution with one degree of freedom; lambda_gc_se: standard error of the genomic inflation factor; n_bhr: sample size used for BHR analysis, defined as n_cases + n_controls for VTE.

File Name: Supplementary Data 9
Description: LOVO results for variants from significant collapsing associations with VTE.

P values shown are two-sided and no adjustments were made for multiple comparisons. Significant associations were highlighted in red. Region: gene name; Group: annotation mask; max_MAF: maximum MAF cutoff; Pvalue: p value for SKAT-O test; Pvalue_Burden: p value for BURDEN test; Pvalue_SKAT: p value for SKAT test; BETA_Burden: effect size of gene-level collapsing analysis; SE_Burden: standard error of BETA_Burden; MAC: minor allele count in the set; MAC_case: minor allele count in cases; MAC_control: minor allele count in controls; Number_rare: number of markers that are not ultra-rare with MAC>10; Number_ultra_rare: number of markers that are ultra-rare with MAC≤10.

File Name: Supplementary Data 10
Description: Variant-level analyses of identified influential variants within PHPT1 and SRSF6 using Firth's bias-reduced logistic regression in the 14,723 VTE cases and 334,315 control subjects. P values shown are two-sided and no adjustments were made for multiple comparisons. Region: gene name; AC_Allele2: allele count of allele 2; AF_Allele2: allele frequency of allele 2; MissingRate: missing rate; AF_case: allele frequency of allele 2 in cases; AF_ctrl: allele frequency of allele 2 in controls; N_case: sample size in cases; N_ctrl: sample size in controls; sample sizes with different genotypes (heterozygous / homozygous) in cases and controls

File Name: Supplementary Data 11
Description: Odds ratio of VTE by PRS_{GW} category and rare coding variant carrier status using Logistic regression models, with covariates including age, sex, and 10 PCs. P values shown are two-sided and no adjustments were made for multiple comparisons.

File Name: Supplementary Data 12
Description: Association between rare coding variant carrier status and VTE stratified by PRS_{GW} quintiles. The prevalence of VTE between rare variants carriers and noncarriers within each quintile were compared. Statistical difference was tested using logistic regression models with covariates including age, sex, and 10 PCs. P values shown are two-sided and no adjustments were made for multiple comparisons.

File Name: Supplementary Data 13
Description: Multiplicative interaction between PRS_{GW} category and rare coding variant carrier status for VTE. The multiplicative interaction effects were measured by adding an interaction term of continuous PRS_{GW} and rare variants carrier status in logistic regression models.

File Name: Supplementary Data 14
Description: Additive interaction between PRS_{GW} category and rare coding variant carrier status for VTE. Additive interaction effects were measured as the RERI (relative excess risk due to interaction), S (synergy index), and AP (attributable proportion) due to interaction using epiR package, with dichotomous categories for standardized PRS_{GW} employed: low (<0) and high (>0) risk.

File Name: Supplementary Data 15
Description: Interaction between Factor V Leiden and rare coding variant carrier status for VTE. The multiplicative interaction effects were measured by adding an interaction term of continuous PRSGW and rare variants carrier status in logistic regression models. The additive interaction effects were measured as the RERI (relative excess risk due to interaction), S (synergy index), and AP (attributable proportion) due to interaction using epiR package

File Name: Supplementary Data 16
Description: Survival association between identified coding variant genes and incident VTE. HR and 95%CI was calculated through CPH regression in participants without VTE at baseline (6,920 incident VTE cases and 293,603 controls) and adjusted for age, sex, and the first 10 PCs. Bonferroni correction were applied respectively for the number of significant genes or lead SNPs in that particular analysis and P values after Bonferroni correction were provided.

File Name: Supplementary Data 17
Description: Gene set enrichment analysis results of VTE associated genes using FUMA. P values shown are two-sided and adjP were adjusted for multiple testing by the Bonferroni approach.
GeneSet: Label of samples; N_genes: The number of significantly regulated genes of a given category and label; N_overlap: Number of input genes overlapping with significantly regulated genes of a given category and label; p: Hypergeometric test P-value; adjP: Bonferroni corrected P-value; genes: Input genes overlapping with significantly regulated genes of a given category and label.

File Name: Supplementary Data 18
Description: GTEx tissue specificity test results of VTE associated genes using FUMA. P values shown are two-sided and adjP were adjusted for multiple testing by the Bonferroni approach.
GeneSet: Label of tissues; N_genes: The number of significantly regulated genes of a given tissue; N_overlap: Number of input genes overlapping with significantly regulated genes of a given tissue; p: Hypergeometric test P-value; adjP: Bonferroni corrected P-value; genes: Input genes overlapping with significantly regulated genes of a given tissue.

File Name: Supplementary Data 19
Description: Details for predifined phenotypes in PheWAS analysis.

File Name: Supplementary Data 20
Description: PheWAS results for VTE-associated genes identified through WES analysis.
P values shown are two-sided and “Bonferroni corrected P” were adjusted for multiple testing by the Bonferroni approach. P values highlighted in bold are nominally significant, with red highlight significant results after Bonferroni correction.
Abbreviations: RBC, Red blood cell count; HGB, hemoglobin concentration; HCT, Hematocrit percentage; MCV, Mean corpuscular volume; WBC, White blood cell count; BASO, Basophil count; EO, Eosinophil count; MONO, Monocyte count; NEUT, Neutrophil count; LYMPH, Lymphocyte count; MPV, Mean platelet volume; PLT, Platelet count; APOA, Apolipoprotein A;

APOB, Apolipoprotein B; CHOL, Cholesterol; CRP, C-reactive protein; GLU, Glucose; HBA1C, Glycated hemoglobin; LDLD, LDL direct; LPA, Lipoprotein A; TRIG, Triglycerides; IGF1, IGF-1; ALB, Albumin; ALT, Alanine aminotransferase; AST, Aspartate aminotransferase; BILD, Direct bilirubin; GGT, Gamma glutamyltransferase; TBIL, Total bilirubin; F11R, Junctional adhesion molecule A; F2R, Proteinase-activated receptor 1; F3, Tissue factor; F7, Coagulation factor VII; F9, Coagulation factor IX; VWF, von Willebrand factor.
