# Supplementary material for: Genetic associations of protein-coding variants in venous thromboembolism
Source: Nat Commun. 2024 Apr 1;15:2819. doi: 10.1038/s41467-024-47178-8 (PMC10984941; doi:10.1038/s41467-024-47178-8)
Supplement: Supplementary file 5 — Reporting Summary [file 41467_2024_47178_MOESM5_ESM.pdf]

nature portfolio

Corresponding author(s): Jin-Tai Yu, Qiang Dong, Wei Cheng

Last updated by author(s): Feb 25, 2024

Reporting Summary

Nature Portfolio wishes to improve the reproducibility of the work that we publish. This form provides structure for consistency and transparency in reporting. For further information on Nature Portfolio policies, see our Editorial Policies and the Editorial Policy Checklist.

Statistics

For all statistical analyses, confirm that the following items are present in the figure legend, table legend, main text, or Methods section.

n/a	Confirmed	
☐	☒	The exact sample size (*n*) for each experimental group/condition, given as a discrete number and unit of measurement
☐	☒	A statement on whether measurements were taken from distinct samples or whether the same sample was measured repeatedly
☐	☒	The statistical test(s) used AND whether they are one- or two-sided<br>*Only common tests should be described solely by name; describe more complex techniques in the Methods section.*
☐	☒	A description of all covariates tested
☐	☒	A description of any assumptions or corrections, such as tests of normality and adjustment for multiple comparisons
☐	☒	A full description of the statistical parameters including central tendency (e.g. means) or other basic estimates (e.g. regression coefficient) AND variation (e.g. standard deviation) or associated estimates of uncertainty (e.g. confidence intervals)
☐	☒	For null hypothesis testing, the test statistic (e.g. *F*, *t*, *r*) with confidence intervals, effect sizes, degrees of freedom and *P* value noted<br>*Give P values as exact values whenever suitable.*
☒	☐	For Bayesian analysis, information on the choice of priors and Markov chain Monte Carlo settings
☒	☐	For hierarchical and complex designs, identification of the appropriate level for tests and full reporting of outcomes
☐	☒	Estimates of effect sizes (e.g. Cohen's *d*, Pearson's *r*), indicating how they were calculated

Our web collection on statistics for biologists contains articles on many of the points above.

Software and code

Policy information about availability of computer code

Data collection	No software was involved in data collection (data used is all directly available from UK Biobank, as described in detail in the paper)
Data analysis	The following software and packages were used for data analysis: FUMA v.1.3.8 (https://fuma.ctglab.nl/), SnpEff v.5.1 (https://pcingola.github.io/SnpEff/), SAIGE-GENE+ v.1.1.6.2 (https://github.com/saigegit/SAIGE), BHR v.0.1.0 (https://github.com/ajaynadig/bhr), PLINK v.2.0 (https://www.cog-genomics.org/plink/), mBAT-combo in GCTA v.1.94.1 (https://yanglab.westlake.edu.cn/software/gcta/#mBAT-combo), METAL v.2011-03-25 (http://csg.sph.umich.edu/abecasis/Metal/) and R v.4.2.0 (https://www.r-project.org/).

For manuscripts utilizing custom algorithms or software that are central to the research but not yet described in published literature, software must be made available to editors and reviewers. We strongly encourage code deposition in a community repository (e.g. GitHub). See the Nature Portfolio guidelines for submitting code & software for further information.

Data

Policy information about availability of data

All manuscripts must include a data availability statement. This statement should provide the following information, where applicable:

- Accession codes, unique identifiers, or web links for publicly available datasets
- A description of any restrictions on data availability
- For clinical datasets or third party data, please ensure that the statement adheres to our policy

Individual level data from the UKB samples are available through UKB (https://www.ukbiobank.ac.uk/) under application number 19542. FinnGen GWAS summary statistics are publicly accessible (http://r8.FinnGen.fi). Source data are provided with this paper.

Research involving human participants, their data, or biological material

Policy information about studies with human participants or human data. See also policy information about sex, gender (identity/presentation), and sexual orientation and race, ethnicity and racism.

Reporting on sex and gender	We took sex into considerations in our study and our findings could apply to both male and female. Sex in the UK Biobank was determined based on self-reporting data via questionnaire, and all included participants gave written informed consent for sharing of individual-level data.
Reporting on race, ethnicity, or other socially relevant groupings	Ethnic background (Field ID 21000) and genetic ethnic grouping (Field ID 22006) was used to define ethnic backgrounds in this study. We included the White British samples with "Caucasian" genetic ethnic grouping in the main analysis and also conducted ancestry-specific and cross-ancestry meta-analysis to investigate the generalizability of our findings.
Population characteristics	A total of 349,038 unrelated White British samples with "Caucasian" genetic ethnic grouping in the UKB were included in the WES analyses, which included 14,723 VTE cases and 334,315 controls. The median age at enrollment was 58.0 years, and 161,319 participants (53.8%) were women (more demographic and clinical characteristics of the study population by VTE case-control status were provided in Supplementary Table 1)
Recruitment	The UKB enrolled the participants aged 40-69 years between 2006 and 2010 for baseline assessments in 22 centers across the UK. The assessment visits comprised interviews and questionnaires covering lifestyles and health conditions, physical measures, biological samples, imaging, and genotyping. The database is linked to national health datasets, including primary care, hospital inpatient, death, and cancer registration data.
Ethics oversight	UK Biobank has received ethical approval from the National Health Service National Research Ethics Service and all participants gave informed consent through electronic signatures. This study was performed based on application number 19542.

Note that full information on the approval of the study protocol must also be provided in the manuscript.

Field-specific reporting

Please select the one below that is the best fit for your research. If you are not sure, read the appropriate sections before making your selection.

☒ Life sciences ☐ Behavioural & social sciences ☐ Ecological, evolutionary & environmental sciences

For a reference copy of the document with all sections, see nature.com/documents/nr-reporting-summary-flat.pdf

Life sciences study design

All studies must disclose on these points even when the disclosure is negative.

Sample size	No statistical methods were used to predetermine sample sizes. All currently available sample in the UK Biobank were included.
Data exclusions	Participants without whole-exome sequencing data, those failed to pass quality control, individuals with diagnosis of superficial or unclear site of thrombophlebitis (I80.0, I80.3, I80.8, I80.9), portal vein thrombosis (I81), BuddChiari syndrome (I82.0), and known coagulation defects, were excluded.
Replication	For external replication of our identified signals in an independent sample cohort, we used summary statistics from the FinnGen Consortium online results (version 8), which included 17,048 VTE cases and 325,451 controls. Of the six identified genes, five replicated when searching for the most significant variants mapped to each gene, while four were validated by our 'mBAT-combo' gene-based analysis (PBonferroni<0.05). Furthermore, of the 13 identified lead SNPs, 12 were replicated.
Randomization	In this exome study, age, sex, and the first 10 genetic principal components (calculated with whole-exome sequencing data) were adjusted.
Blinding	Blinding was not applicable to this study as this study is observational.

Reporting for specific materials, systems and methods

We require information from authors about some types of materials, experimental systems and methods used in many studies. Here, indicate whether each material, system or method listed is relevant to your study. If you are not sure if a list item applies to your research, read the appropriate section before selecting a response.

Materials & experimental systems

n/a	Involved in the study
☒	☐ Antibodies
☒	☐ Eukaryotic cell lines
☒	☐ Palaeontology and archaeology
☒	☐ Animals and other organisms
☒	☐ Clinical data
☒	☐ Dual use research of concern
☒	☐ Plants

Methods

n/a	Involved in the study
☒	☐ ChIP-seq
☒	☐ Flow cytometry
☒	☐ MRI-based neuroimaging

Plants

Seed stocks	*Report on the source of all seed stocks or other plant material used. If applicable, state the seed stock centre and catalogue number. If plant specimens were collected from the field, describe the collection location, date and sampling procedures.*
Novel plant genotypes	*Describe the methods by which all novel plant genotypes were produced. This includes those generated by transgenic approaches, gene editing, chemical/radiation-based mutagenesis and hybridization. For transgenic lines, describe the transformation method, the number of independent lines analyzed and the generation upon which experiments were performed. For gene-edited lines, describe the editor used, the endogenous sequence targeted for editing, the targeting guide RNA sequence (if applicable) and how the editor was applied.*
Authentication	*Describe any authentication procedures for each seed stock used or novel genotype generated. Describe any experiments used to assess the effect of a mutation and, where applicable, how potential secondary effects (e.g. second site T-DNA insertions, mosiacism, off-target gene editing) were examined.*
